# The Anti-Inflammatory Effects of Adipose Tissue Mesenchymal Stem Cell Exosomes in a Mouse Model of Inflammatory Bowel Disease

**DOI:** 10.3390/ijms242316877

**Published:** 2023-11-28

**Authors:** Jun Ho Lee, Jan Lötvall, Byong Seung Cho

**Affiliations:** 1 ExoCoBio Exosome Institute (EEI), ExoCoBio Inc., STE 306, 19 Gasan digital 1-ro, Geumcheon-gu, Seoul 08594, Republic of Korea; junho.lee@exocobio.com (J.H.L.); ceo@exocobio.com (B.S.C.); 2 Krefting Research Centre, Institute of Medicine, University of Gothenburg, 40530 Göteborg, Sweden

**Keywords:** exosome, extracellular vesicles, mesenchymal stem cells, anti-inflammation, inflammatory bowel disease (IBD)

## Abstract

Inflammatory bowel disease (IBD) is a group of chronic, relapsing inflammatory disorders that affect the gastrointestinal tract, with the primary subtypes being ulcerative colitis (UC) and Crohn’s disease (CD). We aimed to evaluate the therapeutic potential of extracellular vesicles released by adipose-tissue-derived mesenchymal stem cells, which we, in this manuscript, call “exosomes” (ASC-EXOs), in a mouse model of IBD. We specifically aimed to determine the effectiveness of different treatment protocols and compare the effects with that of anti-IL-12 p40 monoclonal antibody. The addition of dextran sulfate sodium (DSS) to drinking water induced multiple signs of IBD, including weight loss, soft stool, and bloody feces. ASC-EXOs given by either intraperitoneal (IP) or intravenous (IV) routes resulted in moderate improvement in these signs of IBD. IV ASC-EXOs resulted in significantly reduced body weight loss, improved histopathological scoring, and suppressed the disease activity index (DAI) compared to the IBD control group. Also, a reduction in PCR for pro-inflammatory cytokines was observed. IV ASC treatment resulted in dose-related reduction in IBD signs, including weight loss. An increasing number of injections with ASC-EXOs reduced histopathological scores as well as DAI. Co-administration of ASC-EXOs with anti-IL-12 p40 significantly decreased DAI scores in the ASC-EXO + anti-IL-12 p40 group. In conclusion, ASC-EXOs have potential as a therapeutic agent for IBD, but the route of administration, number of injections, and dosage need to be considered to optimize the effects of ASC-EXO treatment. This study also highlights the potential benefits of combination therapies of ASC-EXOs and anti-IL-12. Our findings pave the way for further studies to unravel the underlying therapeutic mechanisms of ASC-EXOs in IBD treatment.

## 1. Introduction

Inflammatory bowel disease (IBD) is a group of chronic, relapsing inflammatory disorders that affects the gastrointestinal tract, primarily consisting of the subtypes ulcerative colitis (UC) and Crohn’s disease (CD) [1,2,3,4]. Various symptoms can be caused by IBD, such as abdominal pain, diarrhea, weight loss, and fatigue, followed by periods of remission [3,4]. These diseases result in significant morbidity beyond the direct effects of the disease, including complications such as strictures, fistulas, and an increased risk of colorectal cancer [1,3,4]. The pathogenesis of IBD is complex and multifactorial, but dysfunction of the immune system is related to IBD [1,2]. Conventional treatments for IBD, such as aminosalicylates, corticosteroids (CS), immunomodulators, biologics, and oral small molecules primarily focus on controlling inflammation and inducing and maintaining remission to improve the life quality of patients [5,6]. However, these treatments have limitations, such as adverse effects, limited efficacy, and a high risk of disease relapse [5,7]. Thus, there is a need for novel therapeutic approaches with improved safety and sustained efficacy in IBD management. One such future drug opportunity is anti-inflammatory exosomes, derived from mesenchymal stem cells [6].

Mesenchymal stem cells (MSCs) can be isolated from various origins, such as bone marrow, umbilical cords, and adipose tissue. MSCs have been explored for over two decades as a cell therapy with functions of regeneration and immunomodulation in a multitude of diseases [8]. Extracellular vesicles released by MSCs, also called exosomes, can induce regeneration and immunomodulatory effects in multiple tissues and several disease models [9]. Exosomes derived from umbilical cord or bone marrow MSC attenuate inflammation and improve mucosal barrier repair in DSS-induced colitis [10]. All cells have the capacity to release nano-sized extracellular vesicles (30–150 nm), and these vesicles contribute intricately to cell-to-cell communication through the transfer of proteins, lipids, and nucleic acids from one cell to another [11,12]. In this paper, we have decided to use the term “exosome” to represent any vesicle released by ASCs. With the features of exosomes, they can be used instead of cells to treat several diseases. Indeed, adipose tissue mesenchymal stem cell exosomes (ASC-EXO) have shown efficacy in a vast variety of inflammatory disorders in many organs, including the heart, lung, liver, kidney, and gastrointestinal tract. These exosomes contain multiple proteins, lipids, and RNA species, which can modulate the complex interaction between immune cells and the intestinal mucosa during IBD pathogenesis [13]. Moreover, the anti-inflammatory and immunosuppressive effects of MSC exosomes may help in reducing the severity of IBD by reducing the excessive immune response and promoting a balanced immune environment. MSC exosomes have, therefore, gained attention for their potential therapeutic effects in IBD and have become a promising candidate for IBD treatment [12]. The exact mode of action of MSC-derived exosomes remains obscure, but arrays of molecules are likely to be involved, including cargo proteins, cargo RNA, and membrane proteins of the exosomes [14].

It is not known which protocol of administration of exosomes would be most efficient in treating IBD. The aim of this study is, therefore, to evaluate the efficacy specifically of ASC-EXOs given via different routes, with different frequencies, and in different doses in models of acute IBD. Any observed efficacy was also tested in the chronic model of the disease. As anti-IL-12 antibodies have shown some efficacy in IBD models, we also asked whether ASC-EXOs can enhance efficacy when added to anti-IL-12 for IBD treatment. We further aimed to explore the putative mechanisms by which ASC-EXOs affect anti-inflammation and immunomodulation.

## 2. Results

### 2.1. Effects of ASC-EXO IV/IP Injection on an Acute IBD Model

The careful characterization of ASC-EXOs has been previously published, including the identification of tetraspanins using FACS of Dynabeads specific for CD9, CD63, and CD81, size and numbers using nano-tracking analysis, and negative stain electron microscopy [15]. The addition of 2% DSS into drinking water induced multiple signs of IBD, including weight loss, soft stool, and blood in feces. One challenged and untreated mouse died during this experiment. Repeated injections of ASC-EXOs injected IP (1 × 10^10^) or IV (1 × 10^10^) once a day for ten days resulted in moderate improvement in signs of IBD (Figure S1). No animal died in the active treatment groups.

The ASC-EXO IV group lost less weight during the experiment compared to the vehicle IBD control group on days 5 to 10 after treatment initiation. Also, the group treated with ASC-EXOS lost less weight during the experiment compared to the vehicle IBD control group on days 5 to 7 after treatment initiation, with a tendency for IV treatment being more effective than IP treatment (Figure S1B). Considering the clinical scoring based on bleeding, stool consistency, and weight loss patterns, the DAI score of the ASC-EXO IV group was statistically significantly reduced compared to the untreated IBD control group on days 5 to 10 after treatment initiation (Figure S1C, Table S4). For histopathological scoring, the ASC-EXO IV and IP injection groups showed significantly reduced signs of inflammation compared to the vehicle IBD group (Figure S1D,E). Overall, the data suggest that the IV route of administration of ASC-EXOs is at least as effective as the IP route, which led us to continue our further IBD animal experiment using IV administration.

### 2.2. Effects of ASC-EXO Treatment Frequency on an Acute IBD Model

**Figure 1:**
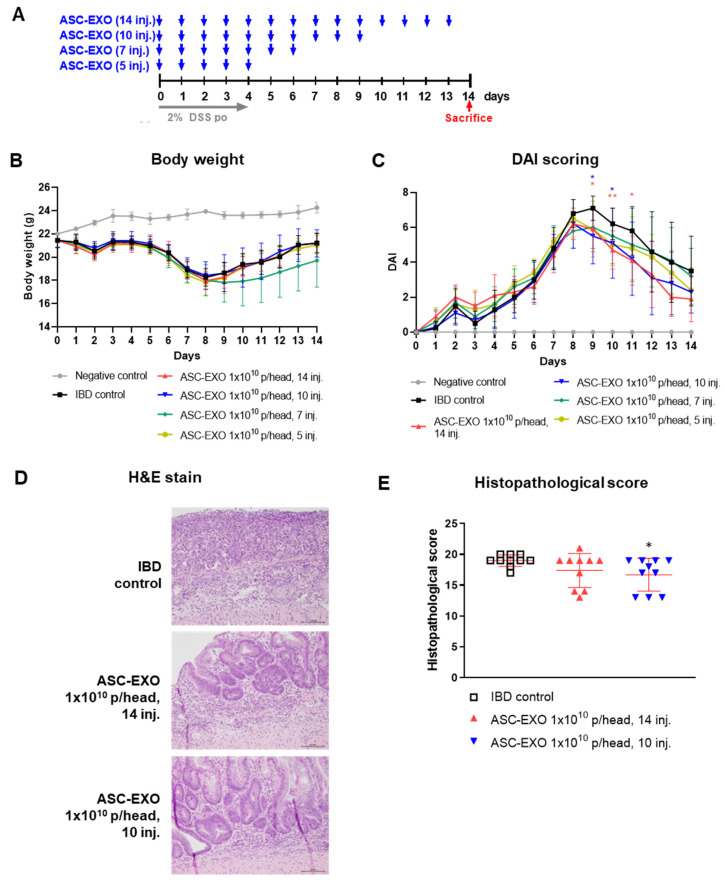
Effects of ASC-EXO treatment frequency on an acute IBD model. A total of 52 mice were divided into a negative control group, IBD control group, and four experimental groups with the negative control group containing two mice, IBD control group containing ten mice, and ASC-EXO treatment group containing ten mice. (**A**) Scheme of study design. Blue arrow means when ASC-EXO is administered and red arrow means the animal sacrifice point. (**B**) Body weight. (**C**) DAI scores of mice were recorded daily from day 0 to 14. Red star means the data with a statistically significant difference between the IBD control and the ASC-EXO 1 × 10^10^, 14 inj. group, and blue star means the data with a statistically significant difference between the IBD control and the 1 × 10^10^, 10 inj. group. (**D**) Large intestine tissue analyzed in H&E staining on day 10 (scale bar = 100 μm). The data are presented as representative images from three independent experiments. (**E**) Histopathological scores. The data are presented as the means ± SD with significance at * *p* < 0.05 and ** *p* < 0.01 compared to IBD control. Abbreviations: Negative control: group without IBD induction; IBD control: IBD induction group; ASC-EXO 1 × 10^10^, 5 inj.: ASC-EXO 1 × 10^10^ particles/head, administered five times; ASC-EXO 1 × 10^10^, 7 inj.: ASC-EXO 1 × 10^10^ particles/head, administered seven times; ASC-EXO 1 × 10^10^, 10 inj.: ASC-EXO 1 × 10^10^ particles/head, administered ten times; ASC-EXO 1 × 10^10^, 14 inj.: ASC-EXO 1 × 10^10^ particles/head, administered fourteen times.

The effects of varying numbers of doses of ASC-EXOs (1 × 10^10^ given IV for five, seven, ten, or fourteen consecutive days) on signs of IBD are shown in Figure 1A. Overall, all regimens of ASC-EXO treatment resulted in a moderate numeric reduction in signs of IBD, including weight loss (Figure 1B). The histopathological scoring of animals treated for ten and fourteen days with ASC-EXOs is shown in Figure 1E and shows a moderate reduction in scores in treated mice. For the scoring of other signs of IBD, bleeding was significantly reduced in all ASC-EXO treated groups from day 9 to day 11 and day 12 to day 14. DAI scores were significantly decreased for the groups treated ten or fourteen times, compared to the induced control group from day 9 to day 11, and overall, there was a decreasing trend in DAI score (Figure 1C, Table S1). Histopathological scoring showed improvement in mice treated with ASC-EXOS for ten consecutive days versus the vehicle IBD control group (Figure 1D,E). There were no significant differences in disease DAI scoring in animals treated for five or seven consecutive days.

### 2.3. Dose-Dependent Effects of ASC-EXOs on an Acute IBD Model

**Figure 2:**
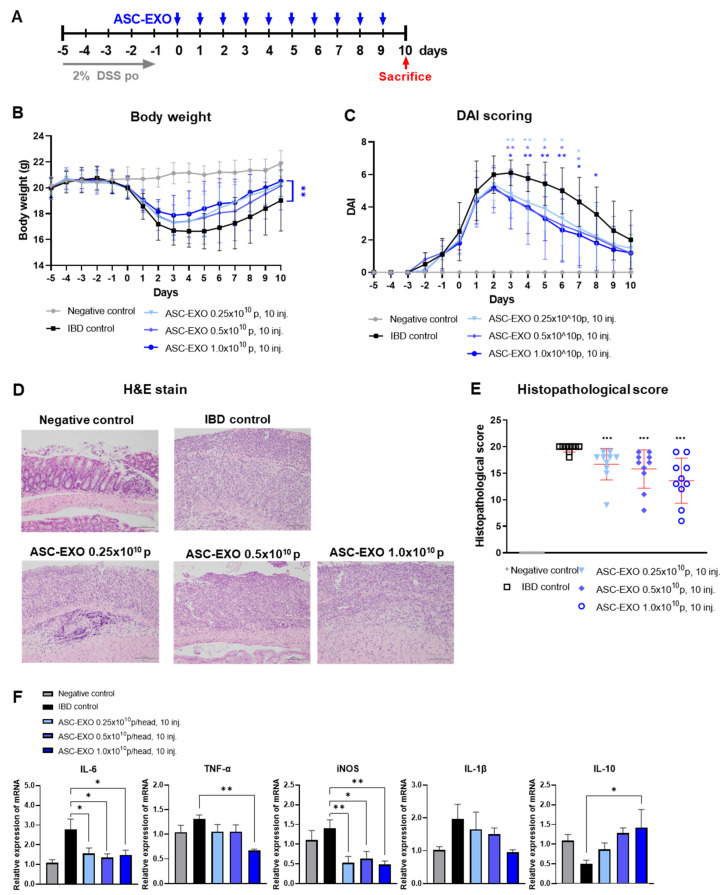
Dose-dependent effects of ASC-EXOs on an acute IBD model. A total of 50 mice were divided into a negative control group, an IBD control group, and three experimental groups, with each group containing ten mice. (**A**) Scheme of study design. Blue arrow means when ASC-EXO is administered and red arrow means the animal sacrifice point. (**B**) Body weight. (**C**) DAI scores of mice were recorded daily from day −5 to 10. (**D**) Large intestine tissue analyzed via H&E staining on day 10 (scale bar = 100 um). The data are presented as representative images from six independent experiments. (**E**) Histopathological score. (**F**) Quantitative real-time PCR for M1 markers IL-6, TNF-α, and iNOS, IL-1β, and M2 marker IL-10 in large intestine tissue (*n* = 5 mice per group). The data are presented as means ± SD with significance at * *p* < 0.05, ** *p* < 0.01, and *** *p* < 0.001 compared to IBD control. Abbreviations: Negative control: group without IBD induction; IBD control: IBD induction group; ASC-EXO 0.25 × 10^10^, 10 inj.: ASC-EXO 0.25 × 10^10^ particles/head, administered ten times; ASC-EXO 0.5 × 10^10^, 10 inj.: ASC-EXO 0.5 × 10^10^ particles/head, administered ten times; ASC-EXO 1 × 10^10^, 10 inj.: ASC-EXO 1 × 10^10^ particles/head, administered ten times.

In the next experiment, we determined the dose-dependency of treatment with ASC-EXOs (0.25 × 10^10^, 0.5 × 10^10^, and 1 × 10^10^) in IBD. In the vehicle IBD control group, one mouse died on day 3. All animals in which IBD was induced started losing weight immediately after the addition of 2% of DSS in drinking water, compared to mice given normal water. Treatment with all doses of ASC-EXOs in IBD mice numerically reduced the loss in weight from day four (Figure 2A). Body weight measurements demonstrated that from day 5 to the end of the study, the weight levels of all ASC-EXO-treated groups tended to increase compared to the vehicle IBD control (Figure 2B). On day 8, the weight level of the 1 × 10^10^ ASC-EXO group was significantly higher than the vehicle IBD control. In terms of DAI score levels, all ASC-EXO-treated groups displayed a dose-dependent decreasing trend from day 3 post-treatment until the end of the study compared to the vehicle IBD control group (Figure 2C, Table S2). The 1 × 10^10^ ASC-EXO group showed significantly decreased DAI scores from day 3 to 8. Among the DAI scoring sub-evaluation items, stool type scores on day 6 post-treatment were significantly lower for the 1 × 10^10^ ASC-EXO group compared to the vehicle IBD control group. Based on the histopathological examination of the colon tissue, we observed that the levels of all histopathological categories were decreased in an ASC-EXO dose-dependent manner, such that total histological scores were significantly reduced in the three ASC-EXO groups compared to the vehicle IBD control group. Notably, the 1 × 10^10^ ASC-EXO group showed significantly decreased scores in the extent of inflammation, infiltration neutrophils + lympho-histiocytes, extent of crypt damage, loss of goblet cells, and reactive epithelial hyperplasia compared to the vehicle IBD control group. (Figure 2D,E). The ASC treatment groups showed significant downregulation of mRNA for IL-6, TNF-α, and iNOS, which are all known to be involved in intestinal inflammation (Figure 2F). ASC-EXO dose-dependently increases the expression of mRNA for IL-10 in tissues.

### 2.4. Co-Administration Effect of ASC-EXOs with an Antibody Drug on an Acute IBD Model

**Figure 3:**
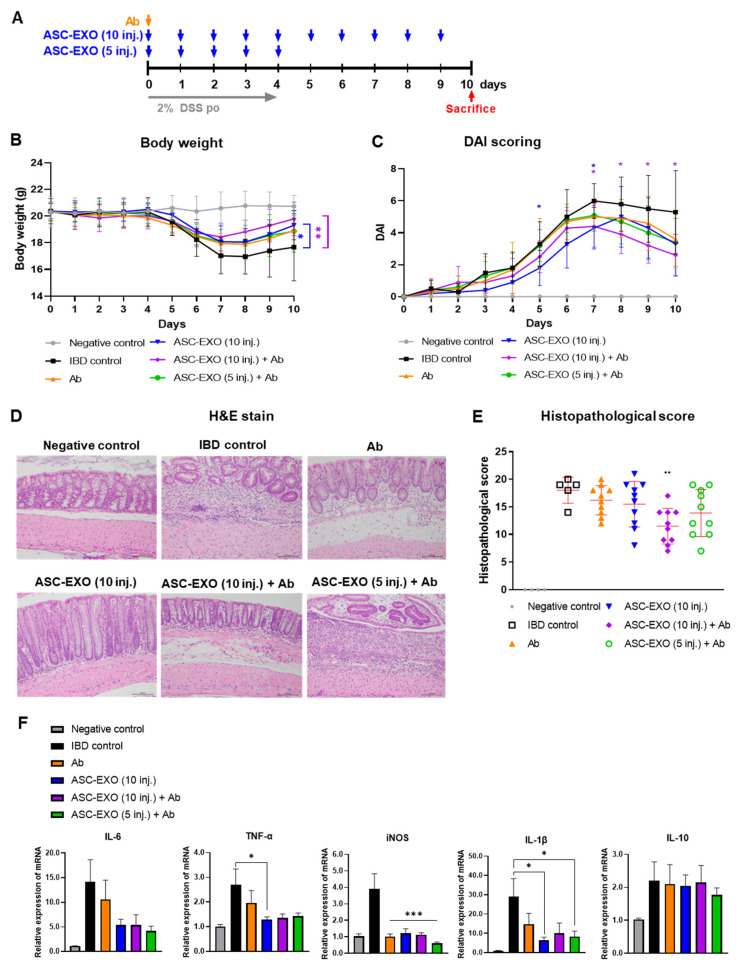
Co-administration effect of ASC-EXOs with anti-IL-12 p40 on an acute mouse model. A total of 50 mice were divided into a negative control group, an IBD control group, and four experimental groups, with the negative control containing four mice, IBD control group containing six mice, and each experimental group containing ten mice. (**A**) Scheme of study design. Blue arrow means when ASC-EXO is administered, orange arrow means when Ab (IL-12 p40) is administered, and red arrow means the animal sacrifice point. (**B**) Body weight. (**C**) DAI scores of mice were recorded daily from day 0 to day 10. Blue star means the data with a statistically significant difference between the IBD control and the ASC-EXO 1 × 10^10^, 10 inj. group, and violet star means the data with a statistically significant difference between the IBD control and the1 × 10^10^, 10 inj. + Ab group. (**D**) Large intestine tissue analyzed in H&E staining on day 10 (scale bar = 100 μm). The data are presented as representative images from six independent experiments. (**E**) Histopathological score. (**F**) Quantitative real-time PCR for M1 markers IL-6, TNF-α, iNOS, IL-1β and M2 marker IL-10 in large intestine tissue (*n* = 5 mice per group). The data are presented as the means ± SD with significance at * *p* < 0.05, ** *p* < 0.01, and *** *p* < 0.001 compared to IBD control. Abbreviations: Negative control: group without IBD induction; IBD control: IBD induction group; Ab: anti-IL-12 p40 administration group; ASC-EXO (10 inj.): ASC-EXO 1 × 10^10^ particles/head, administered ten times; ASC-EXO (5 inj.), ASC-EXO 1 × 10^10^ particles/head, administered five times.

Upon observation of general symptoms, one mouse in the vehicle IBD control group died on day 8 after inducing acute IBD. The cause of death is unclear. Treatment was initiated with the ASC-EXO combination with anti-IL-12 p40 (Ab) in IBD mice (Figure 3A). Body weight measurements demonstrated that from day 7 to the end of the study, the weight levels of all ASC-EXO-treated groups tended to increase compared to the vehicle IBD control (Figure 3B). The DAI score was significantly reduced from day 7 to day 10 in the group treated with the combination of 1 × 10^10^ ASC-EXO and anti-IL-12 p40 (Figure 3C, Table S3). Specifically, the bleeding levels were reduced compared to the vehicle IBD control group. Further, body weight loss was significantly decreased from day 7 to day 10 in the group treated with the combination of 1 × 10^10^ ASC-EXO and anti-IL-12 p40. No significant differences in stool type levels were observed between any groups. The composite histopathological score was significantly reduced in the group treated ten times with the combination of ASC-EXOs and anti-IL-12 p40 compared to the vehicle IBD control group. All treated groups exhibited a trend of lower histopathologic scoring levels compared to the untreated vehicle IBD control group, but no significant differences were observed (Figure 3D,E). Both ASC-EXOs and combined ASC-EXOs with anti-IL-12 p40 showed significantly downregulated mRNA expression levels of genes encoding IL-6, TNF-α, and iNOS, which are known to be involved in intestinal inflammation (Figure 3F). Anti-IL-12 p40 tended to decrease iNOS mRNA more than ASC-EXOs alone.

### 2.5. Administration Effect of ASC-EXOs on a Chronic IBD Model

Extended DSS given in repeated cycles is considered to be a model of chronic IBD and is associated with significant mortality. ASC-EXOs (1 × 10^10^ or 0.5 × 10^10^) given IV were associated with significantly increased survival compared to the untreated chronic IBD control group (Figure S2B). Only one mouse died in the 1 × 10^10^ ASC-EXO group, whereas four mice died in the 0.5 × 10^10^ ASC-EXO group and six mice in the untreated vehicle IBD control group. There were no significant differences in body weight and DAI scores among the groups (Figure S2C,D).

## 3. Discussion

In this study, we have examined the therapeutic potential of ASC-EXOs for treating IBD using a well-established mouse model where animals are exposed to DSS in drinking water. Multiple ASC-EXO treatment protocols were found to result in reduced signs of disease in the animals, regardless of whether the ASC-EXOs were given by IV or IP administration route. The effect was dependent on the concentration of ASC-EXOs and the number of doses given. The anti-IL-12 antibody drug has previously been shown to reduce signs of IBD, which was observed also in the current experiments. However, ASC-EXOs further reduced the signs of disease when given in addition to anti-IL-12 antibodies. We also tested whether ASC-EXOs have efficacy in a chronic model of IBD and could observe an increased survival rate in this quite severe model. Overall, these results suggest that ASC-EXOs have efficacy in models of IBD.

MSC-derived exosomes have previously been shown to reduce inflammation in IBD models, but different treatment protocols have not been effectively compared. In mouse studies, drugs can easily be given by IP routes, whereas this route is rarely or never used in clinical settings in humans. However, the IP or IV route of administration of exosomes leads to different biodistributions of the exosomes, and, therefore, it is interesting to observe that the efficacy of the ASC-EXOs was more pronounced with IV routing; this is important to consider, as many previous studies have primarily tested IP injection of exosomes in IBD models [15]. Importantly, the finding that IV dosing was more effective than IP dosing probed us to continue any in vivo experimentation using this route.

Increasing the number of treatments with ASC-EXOs further reduced signs of disease compared to fewer doses. Thus, the total dose of ASC-EXOs seems to influence the effects of the disease, and importantly, there are no signs of rebound effects; thus, ASC-EXOs do not lose any efficacy with extended dosing. No further improvement was observed beyond 10 injections. In a previous study, MSC-exosome treatment was administered for seven consecutive days, which improved weight loss, stool type and degree of bleeding. This further underlines the importance of extending the therapeutic regimen with exosome therapeutics when aiming to manage severe diseases such as IBD [16].

We also compared the effects of three increasing doses of ASC-EXO (0.25 × 10^10^, 0.5 × 10^10^, and 1 × 10^10^) given IV to the IBD model. Importantly, dose-response-related effects were observed on body weight, DAI scoring, and histopathological scoring. Overall, this supports the conclusion that the total quantity of ASC-EXOs is important for the efficacy of this disease model. These series of experiments also added detailed mechanistic information, as the PCRs for pro- and anti-inflammatory molecules were analyzed in tissue samples from the colon of treated mice. Thus, both IL-6 and iNOS were efficiently inhibited with the lowest dose of ASC-EXOs, whereas a tendency of dose-related inhibition was observed on the pro-inflammatory cytokines IL-1β and TNF-α. Importantly, a dose-related increase in expression of the anti-inflammatory cytokine IL-10 was observed in tissues, which implies that some of the anti-inflammatory effects of the ASC-EXOs may be mediated by IL-10, which previously has been shown for severe inflammation models using other types of MSC vesicles [13,17].

It has previously been shown that blocking IL-12 reduces disease severity in mouse models of IBD. In the current experiments, we observed that co-treatment with ASC-EXOs and an anti-IL-12 p40 antibody drug resulted in a greater reduction in disease signs compared to the antibody alone. Thus, it is unlikely that the ASC-EXO effects are dependent on the influence of the IL-12 inflammation axis. It is possible that combinations of blockage of IL-12 and treatment with ASC-EXOs would result in increased clinical efficacy, but to confirm this, extensive additional experimentation would be required.

In addition to the observations that ASC-EXOs show anti-inflammatory efficacy in the acute IBD mouse model, our early results from a chronic IBD model support the concept that ASC-EXO treatment has effects in more prolonged disease, even showing a reduction in mortality. This observation is important considering the chronic progression of IBD, which necessitates effective long-term therapeutic strategies. The potential of ASC-EXOs in improving survival rates, as suggested by our findings, aligns with the ongoing research efforts to develop long-lasting therapeutic strategies for chronic diseases such as IBD [18].

Our results start to unveil some of the mechanisms by which ASC-EXOs convey anti-inflammatory function in vivo. Thus, PCR of tissues suggests that ASC-EXOs reduce local production of pro-inflammatory cytokines such as IL-6, TNF-α, IL-1beta, and iNOS, supporting a general effect of the exosomes [18]. In contrast, the anti-inflammatory cytokine IL-10 was increased, suggesting an activation of T-regulatory cells in the tissue [18,19]. The effects of ASC-EXOs are similar to what can be observed with whole-cell mesenchymal stem cell treatments in different disease models [20].

Determining whether these pre-clinical data are relevant for clinical disease remains unclear. Firstly, the models that have been used in this study utilize DSS in drinking water, which may induce inflammatory processes that are different from those present in clinical cases of IBD. However, as anti-IL-12 p40 antibody drug treatment has clinical efficacy, and is effective in this model, we suggest that the model may have some predictive value. The efficacy of ASC-EXOs in patients with IBD needs to be validated in large clinical trials. The suitability of exosome-based therapeutics in these diseases may, however, be questioned, as the treatment may need to be given over extended periods of time. Importantly, manufacturing of pharmaceutical grade exosomes remains costly, which provides another hurdle to provide cost-effective treatments for these diseases.

In summary, our research supports the concept that ASC-EXOs have efficacy in treating IBD. Our results highlight the importance of various factors, such as administration route, number of injections, and dosage in maximizing the therapeutic potential of ASC-EXOs. Our findings also point towards the potential benefits of combination therapies and the need for long-term therapeutic strategies, in which ASC-EXOs add anti-inflammatory efficacy to other treatments. Overall, this study sets the stage for future studies to unravel the underlying mechanisms of ASC-EXOs’ therapeutic effects for application in IBD treatment and potentially testing ASC-EXOs in clinical disease.

## 4. Materials and Method

### 4.1. Generation of ASCs in Conditioned Media (CM)

Human adipose tissues from a healthy donor were collected by ExoCoBio Inc. (Seoul, Republic of Korea) with the approval of the Institutional Review Board of CHA University Medical Center (Seoul, Republic of Korea) and assessed according to guidelines of the Korean Ministry of Food and Drug Safety (MFDS), and as described previously [21]. Briefly, ASCs isolated from the adipose tissue were sub-cultured at a density of 3000 cells/cm^2^ in MEM-α (Gibco, Grand Island, NY, USA) containing 10% fetal bovine serum (FBS) (Thermo Fisher Scientific, Carlsbad, CA, USA). Cell stocks of passage 2 were stored in liquid nitrogen (1.1 × 10^6^ cells/mL/vial). The quality of the ASCs was assessed by testing for cell viability, sterility, and the presence of mycoplasma and endotoxin. The MSC surface markers for ASCs were determined by the flow cytometry of CD29, CD73, CD105, and CD146. Trilineage differentiation (adipogenic, chondrogenic, and osteogenic differentiation) abilities of ASCs were also determined using standard procedures. To generate the ASC-CM, a cell stock was thawed and cultured until passage 7. ASCs at passage 7 were plated at a density of 6000 cells/cm^2^ and cultured with MEM-α containing 10% FBS in a humidified atmosphere of 5% CO_2_ in air at 37 °C for 3 days up to 90% confluency. For the collection of exosomes, cells were washed three times with PBS and supplemented with phenol-red-free MEM-α. The cells were further cultured for 24 h before the ASC-CM was collected for further exosome isolation [15,22]. 

### 4.2. ASC-EXO Isolation

ASC-derived exosomes (ASC-EXOs) were isolated from the ASC-CM by tangential flow filtration (TFF) as previously described. Firstly, the CM was filtered through a 0.22-μm polyethersulfone membrane filter (Merck Millipore, Billerica, MA, USA) to remove larger fragments from the CM, such as cells, cell debris, and apoptotic bodies. The CM was then concentrated by tangential-flow filtration with a 500 kDa molecular weight cut-off filter membrane cartridge (GE Healthcare, Chicago, IL, USA), and buffer exchange was performed by diafiltration with PBS. Isolated ASC-EXOS were aliquoted into polypropylene disposable tubes and stored at −80 °C until use. Before experiments, frozen ASC-EXO aliquots were left at 4 °C until completely thawed and were never re-frozen. Further details of the ASC-exosome isolation process can be found in these references: [15,21,22]. Characterization of the ASC-EXOs was performed according to the Minimal Information for Studies of Extracellular Vesicles 2018 (MISEV2018) recommended by the International Society for Extracellular Vesicles. The results of markers for CD9, CD63, and CD81, surface proteins of exosomes, as beads flow cytometry data, which confirm the presence of these tetraspanins, is shown in Figure S3.

### 4.3. Nanoparticle Tracking Analysis (NTA)

To determine the distribution of exosome sizes and concentrations, ASC-EXOS diluted in PBS were analyzed by NTA using a NanoSight NS300 (Malvern Panalytical, Amesbury, UK) equipped with a 642 nm laser [15,21,22]. ASC-EXOs were diluted to provide 20–80 particles per frame; videos were then analyzed by the NTA 3.2 software (Malvern Panalytical, Amesbury, UK). At least 5 videos were captured per sample and >2000 validated tracks were analyzed for each sample. The NTA instrument was regularly monitored for output using 100 nm-sized standard beads (3100A Nanosphere Size Standard; Thermo Fisher Scientific, Waltham, MA, USA).

### 4.4. DSS-Induced IBD Mouse Model

Acute IBD was induced by orally administering 2% dextran sulfate sodium (DSS; 160110; MP Biomedicals, Solon, OH, USA) in drinking water from day 0 to day 4 to 6-week-old C57BL/6 mice. The chronic IBD model was induced by cyclically administering 2% DSS in drinking water for 5 days during three cycles. Each cycle consisted of DSS administration on days 0–4, 10–14, and 20–24, with regular water provided during the intervening periods with 6-week-old C57BL/6 mice [23].

### 4.5. Route Selection of Administration for an Acute IBD Model

All procedures were approved by the Institutional Animal Care and Use Committee of Knotus, Republic of Korea (IACUC 21-KE-278). A total of 30 mice were divided into one IBD control group and multiple experimental groups, with each group containing ten mice. ASC-exosomes were delivered to each mouse on day 0 to day 9 by intravenous (IV) or intraperitoneal route (IP) at a volume of 200 µL and with ASC-EXO 1 × 10^10^ particles/head. Mice were sacrificed on day 10 for further analysis, including histopathological analysis and scoring (Figure S1A).

### 4.6. Selection Number of Injections for an Acute IBD Model

All procedures were approved by the Institutional Animal Care and Use Committee of Knotus, Republic of Korea (IACUC 21-KE-434). A total of 52 mice were divided into a negative control group, IBD control group, and four experimental groups with the negative control group containing two mice, IBD control group containing ten mice, and ASC-EXO treatment groups containing ten mice. Each treatment group was treated with ASC-EXO 1 × 10^10^ particles at a volume of 200 µL per mouse. ASC-EXO 1 × 10^10^ particles were injected by IV with a different number of injections for each group as follows: 14 times (daily from day 0 to day 13), 10 times (daily from day 0 to day 9), 7 times (daily from day 0 to day 6), and 5 times (daily from day 0 to 4). Mice were sacrificed on day 14 for further analysis, including histopathological analysis and scoring (Figure 1A).

### 4.7. Administration of ASC-EXOs for an Acute IBD Model

All procedures were approved by the Institutional Animal Care and Use Committee of Knotus, Republic of Korea (IACUC 22-KE-0346). A total of 50 mice were divided into a negative control group, IBD control group, and three experimental groups, with each group containing ten mice. Each animal was treated with ASC-EXOs at a volume of 0.2 mL per mouse with 10 injections. The ASC-EXO treatments were administered daily from day 0 to day 9 by IV at a volume of 200 µL. The ASC-EXO treatment groups were injected with different particle numbers for each group as follows: 0.25 × 10^10^, 0.5 × 10^10^, and 1 × 10^10^ particles of ASC-EXOs. Mice were sacrificed on day 10 for further analysis, including histopathological analysis and scoring (Figure 2A).

### 4.8. Co-Administration of ASC-EXOs with an Antibody Drug for an Acute IBD Model

All procedures were approved by the Institutional Animal Care and Use Committee of Knotus, Republic of Korea (IACUC 22-KE-0061). A total of 50 mice were divided into a negative control group, IBD control group, and four experimental groups, with the negative control containing four mice, IBD control group containing six mice, and each experimental groups containing ten mice. Anti-IL-12 p40 (InVivoPlus™ anti-mouse-IL-12 p40, BP0051; Bio X Cell, Lebanon, NH, USA) was administered once on day 0, whereas ASC-EXO treatments were given with different frequencies, but always in a volume of 200 µL per mouse. A volume of 1 × 10^10^ ASC-EXO and anti-IL-12 p40 were injected in different conditions for each group as follows: the anti-IL-12 p40 treatment group, the ten daily ASC-EXO treatments group (daily from day 0 to day 9), the ten ASC-EXO (daily from day 0 to 9) combined with anti-IL-12 p40 injection group, and the five ASC-EXO (daily from day 0 to 4) + anti-IL-12 p40 injection group. Mice were sacrificed on day 14 for further analysis, including histopathological analysis and scoring, as well as measurement of inflammatory markers (Figure 3A).

### 4.9. Administration of ASC-EXOs for a Chronic IBD Model

All procedures were approved by the Institutional Animal Care and Use Committee of Knotus, Republic of Korea (IACUC 22-KE-0347). A total of 40 mice were divided into a negative control group, vehicle IBD control group, and two experimental groups, with each group containing ten mice. The ASC-EXO treatments were administered IV during the DSS administration periods, for a total of 15 injections across the three cycles with a volume of 200 µL per mouse. The ASC-EXO treatment groups were injected with different particle numbers for each group as follows: 0.5 × 10^10^ and 1 × 10^10^ particles of ASC-EXOs. Mice were sacrificed on day 29 for further analysis, including histopathological analysis and scoring (Figure S2A).

### 4.10. Disease Activity Index (DAI) Scoring

The general symptoms of the experimental animals were observed and evaluated based on the disease activity index (DAI) as described by Wang et al. [16]. During experiments, we observed the animals once per day. The observations encompassed a variety of signs, including body weight loss, stool consistency, presence of bleeding, and death, with the onset date and severity of the signs being recorded and scored (Table 1).

### 4.11. Histological Evaluation Method

The histopathological examination was carried out on fixed tissues processed through a series of standard procedures, including trimming, dehydration, paraffin embedding, and sectioning. The specimens were then stained with hematoxylin and eosin (H&E) for examination. Observations were made under an Olympus BX53 light microscope, following the protocols set forth by Wang et al. [18] and Sann H et al. [24]. The histopathological changes observed by the examination are described in Table 2.

### 4.12. Quantitative Polymerase Chain Reaction (qPCR) Assay Method

For gene expression analysis of large intestine tissue, samples were disrupted and homogenized using the TissueLyser II (#85300; Qiagen, MD, USA) by adding stainless steel beads (#69989; Qiagen, MD, USA) for 2 min at 30 Hz. Then, total RNA extraction was performed using the RNeasy Mini Kit (#74104; Qiagen, MD, USA) and complementary DNA (cDNA) was synthesized using the PrimeScript^TM^ RT Master Mix (#RR036A; TaKaRa, Dalian, China) according to the manufacturer’s instructions. The cDNA was analyzed by qPCR assay using specific primers (listed in Table 3) and KAPA SYBR^®^ FAST qPCR Kit Master Mix (#KK4602; Kapa Biosystems, London, UK). Primer sequences are shown in Table 3.

### 4.13. Statistical Analysis

Statistical analysis was conducted using GraphPad Prism 5.0 software (GraphPad, Bethesda, MD, USA). One-way ANOVA was used when comparing more than two groups of samples with one condition, while two-way ANOVA was used for comparisons involving more than two conditions. Post hoc Bonferroni’s correction for multiple comparisons was applied after ANOVA analysis. A significance level of *p* < 0.05 was considered statistically significant, with levels of significance denoted as follows: * *p* < 0.05, ** *p* < 0.01, *** *p* < 0.001, and **** *p* < 0.0001. Data from at least three independent experiments are presented as the mean ± SD.

## Figures and Tables

**Table 1:** DAI scoring assessment.

Score	Body Weight Loss (%)	Stool Type	Bleeding
0	<2%	Normal	No rectal bleeding
1	≥2–<5%	Softer stool	Weak hemoccult
2	≥5–<10%	Moderate diarrhea	Visual blood in stool
3	≥10–<15%	Diarrhea	Fresh rectal bleeding
4	≥15%	-	-

**Table 2:** Histological scoring assessment.

Grade	Extent of Inflammation	Infiltration Neutrophils + Lympho-Histiocytes	Extent of Crypt Damage	Crypt Abscesses	Sub- Mucosal Oedema	Loss of Goblet Cells	Reactive Epithelial Hyperplasia
0	None	None	None	None	None	None	None
1	Mucosa	Focal	Basal one third	Focal	Focal	Focal	Focal
2	Mucosa + submucosa	Multifocal	Basal two thirds	Multifocal	Multifocal	Multifocal	Multifocal
3	Mucosa + submucosa + muscle layer	Diffuse	Entire crypt damage		Diffuse	Diffuse	Diffuse
4	Transmural	-	Crypt damage + ulceration				

**Table 3:** Primer sequences for real-time quantitative PCR analysis of target genes.

Gene	Forward Primer (5′–3′)	Reverse Primer (5′–3′)
*GAPDH*	AAC TTT GGC ATT GTG GAA GG	ACA CAT TGG GGG TAG GAA CA
*IL-6*	GCC AGA GTC CTT CAG AGA GAT ACA	ATT GGA TGG TCT TGG TCC TTA GCC
*TNF-α*	CAG GCG GTG CCT ATG TCT C	CGA TCA CCC CGA AGT TCA GTA G
*iNOS*	GTT CTC AGC CCA ACA ATA CAA GA	GTG GAC GGG TCG ATG TCA C
*IL-1β*	TGC CAC CTT TTG ACA GTG ATG	5AAG GTC CAC GGG AAA GAC AC
*IL-10*	CCA AGC CTT ATC GGA AAT GA	TTT TCA CAG GGG AGA AAT CG

Abbreviations: GAPDH: glyceraldehyde-3-phosphate dehydrogenase; IL-6: interleukin-6; TNF-α: tumor necrosis factor α; iNOS: nitric oxide synthase; IL-1β: interleukin-1β; IL-10: interleukin-10.

## Data Availability

Data is contained within the article and Supplementary Material.

## Supplementary Materials

The supporting information can be downloaded at: https://www.mdpi.com/article/10.3390/ijms242316877/s1.
