# Supplementary material for: The Anti-Inflammatory Effects of Adipose Tissue Mesenchymal Stem Cell Exosomes in a Mouse Model of Inflammatory Bowel Disease
Source: Int J Mol Sci. 2023 Nov 28;24(23):16877. doi: 10.3390/ijms242316877 (PMC10706798; doi:10.3390/ijms242316877)
Supplement: Supplementary file 1 [file ijms-24-16877-s001.zip › ijms-2674235-supplementary.pdf]

Supplementary Information for

Anti-inflammatory effects of adipose tissue mesenchymal stem cell exosomes in mouse model of inflammatory bowel disease

Jun Ho Lee [1], Jan Lötvall[2*] and Byong Seung Cho [1]

[1] ExoCoBio Exosome Institute (EEI), ExoCoBio Inc., STE 306, 19 Gasan digital 1-ro, Geumcheon-gu, Seoul 08594, Republic of Korea
[2] Krefting Research Centre, Institute of Medicine, University of Gothenburg, Sweden
* Correspondence: jan.lotvall@gu.se

This file includes:

- Supplementary Figure 1
- Supplementary Figure 2
- Supplementary Figure 3
- Supplementary Table 1
- Supplementary Table 2
- Supplementary Table 3
- Supplementary Table 4

SUPPLEMENTARY FIGURES

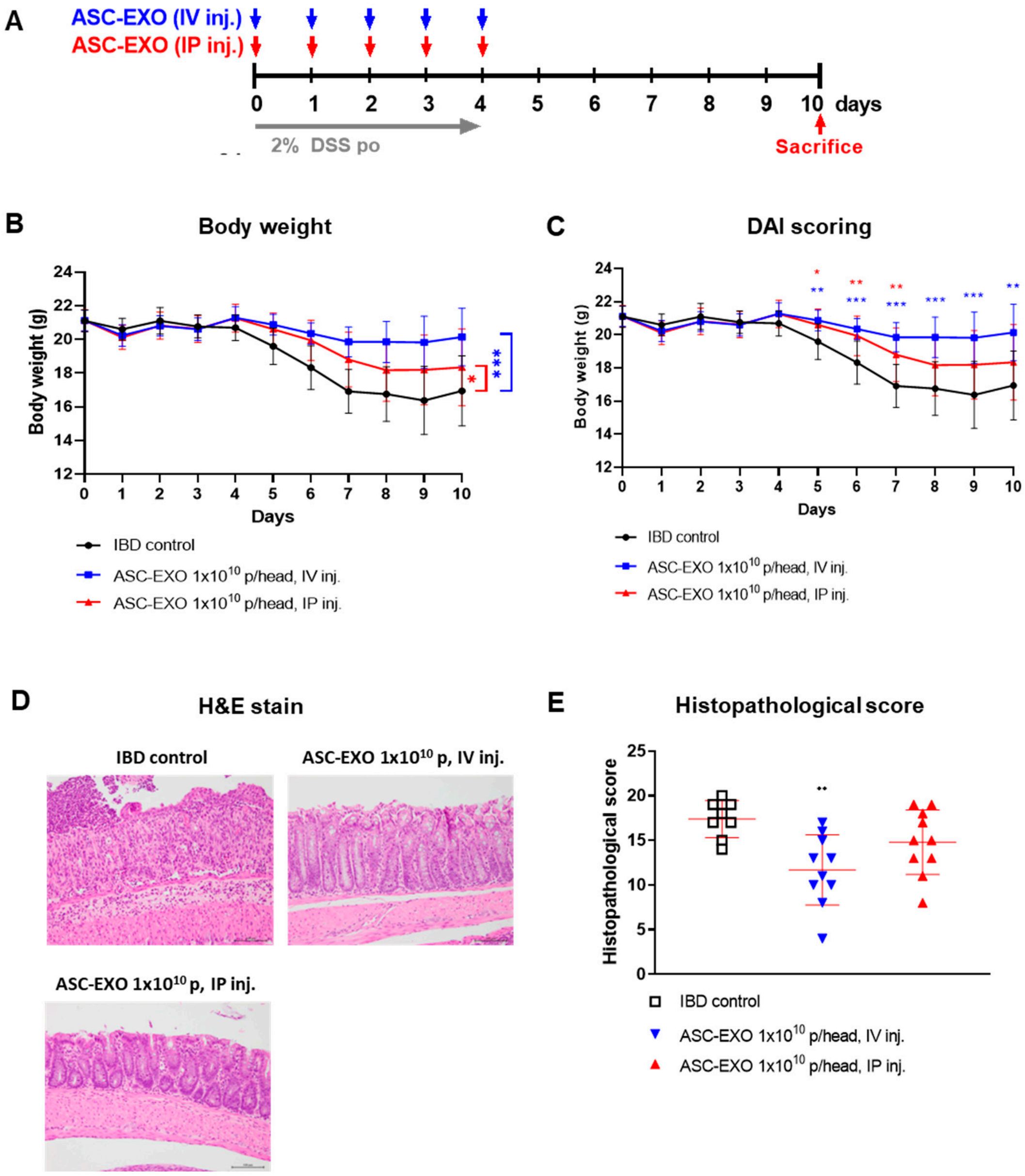

Supplementary figure 1. Effects of ASC-EXO IV/IP injection on acute IBD model. A total of 30 mice were divided into one IBD control group and multiple experimental groups, with each group containing ten mice. (a) Scheme of study design. (b) Body weight (c) DAI score of mice were recorded daily from day 0 to 14. (d) Large intestine tissue analyzed in H&E staining on day 10 (scale bar = 100 µm). The data are presented representative image from three independent experiments. (e) Histopathological score. The data are presented as the means ± SD with significance at *p < 0.05, **p < 0.01 and ***p < 0.001 level compared to IBD control. Abbreviation: IBD control, IBD induction group; ASC-EXO 1 x 10^{10} p/head, IV inj.; ASC-EXO 1 x 10^{10} particles/head, intravenous injection group; ASC-EXO 1 x 10^{10} p/head, IP inj.; ASC-EXO 1 x 10^{10} particles/head, intraperitoneal injection group.

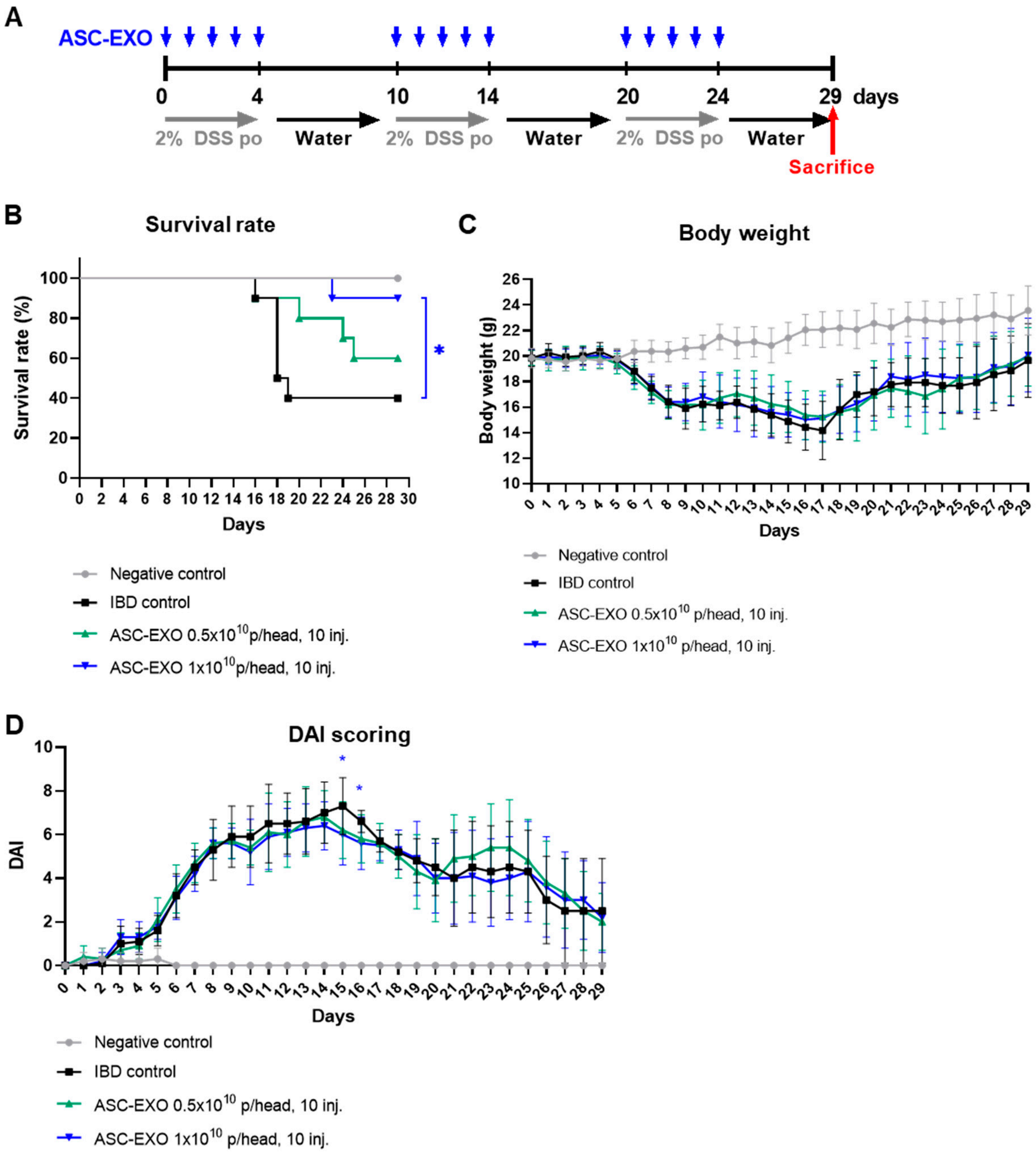

Supplementary figure 2. Administration effect of ASC-EXO on chronic IBD model. A total of 40 mice were divided into negative control group, IBD control group and two experimental groups, with each group containing ten mice. (a) Scheme of study design. (b) Survival rate (c) Body weight (d) DAI score of mice were recorded daily from day 0 to day 29. The data are presented as the means ± SD with significance at *p < 0.05 level compared to IBD control. Abbreviation: IBD control, IBD induction group; ASC-EXO $1x10^{10}$ p/head, IV inj.; ASC-EXO $1x10^{10}$ particles/head, intravenous injection group; ASC-EXO $1.0x10^{10}$ p/head, 10 inj.; ASC-EXO $1x10^{10}$ particles/head, ten injection group.

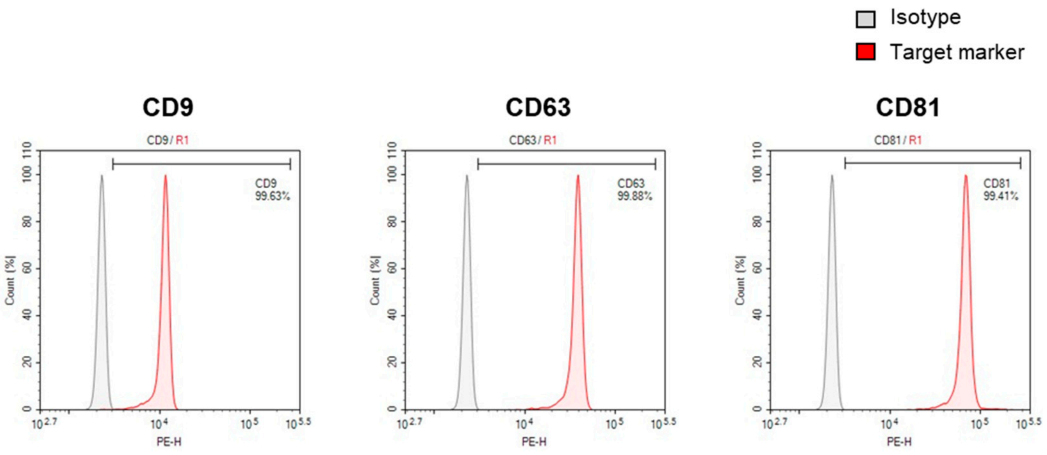

Supplementary figure 3. The exosome surface protein markers (CD9, CD63, CD81) of ASC-EXO. Representative histogram of FCM analysis.

SUPPLEMENTARY TABLES

Supplementary table 1. Effects of ASC-EXO treatment frequency on mouse acute colitis model DAI scoring. DAI score of mice were recorded daily from day 0 to day 14.

Days	DAI Scoring (Average data)					
	Normal	IBD control	ASC-EXO (1 x 10^{10} particles/head, 14 inj.)	ASC-EXO (1 x 10^{10} particles/head, 10 inj.)	ASC-EXO (1 x 10^{10} particles/head, 7 inj.)	ASC-EXO (1 x 10^{10} particles/head, 5 inj.)
Day0	0.0 ± 0.0	0.0± 0.0	0.0± 0.0	0.0± 0.0	0.0± 0.0	0.0± 0.0
Day1	0.0 ± 0.0	0.2± 0.4	0.9± 0.3**	0.3± 0.5	0.6± 0.7	0.6± 0.6
Day2	0.0 ± 0.0	1.5± 1.0	2.0± 0.7	1.1± 0.7	1.7± 0.9	1.6± 1.6
Day3	0.0 ± 0.0	0.5± 0.7	1.5± 0.8*	0.7± 0.7	0.9± 0.7	1.3± 1.3
Day4	0.0 ± 0.0	1.3± 1.1	2.1± 1.2	1.2± 0.9	1.6± 1.0	1.6± 1.6
Day5	0.0 ± 0.0	2.0 ± 0.9	2.3± 0.9	1.9± 1.1	2.6± 1.0	2.8± 2.8
Day6	0.0 ± 0.0	3.0 ± 1.1	2.6± 1.0	2.9± 1.2	3.1± 0.9	3.4± 3.4
Day7	0.0 ± 0.0	4.8 ± 1.2	4.4± 1.0	4.6± 1.0	4.9± 1.2	5.2± 5.2
Day8	0.0 ± 0.0	6.8 ± 0.8	6.2± 0.9	6.2± 1.4	5.8± 1.0	6.5± 6.5
Day9	0.0 ± 0.0	7.1 ± 0.7	5.9± 1.3*	5.5± 1.6*	6.0± 1.2	5.9± 5.9
Day10	0.0 ± 0.0	6.2 ± 0.9	4.7± 1.1**	5.1± 2.0	5.5± 1.6	5.0± 5.0
Day11	0.0 ± 0.0	5.8 ± 1.4	4.1± 1.4*	4.2± 2.1	5.0± 2.1	4.8± 4.8
Day12	0.0 ± 0.0	4.6 ± 2.3	3.3± 1.9	3.1± 2.1	4.6± 2.0	4.3± 4.3
Day13	0.0 ± 0.0	4.0 ± 2.3	2.0± 1.1	2.8± 1.8	4.1± 1.9	3.4± 3.4
Day14	0.0 ± 0.0	3.5 ± 2.0	1.9± 1.3	2.3± 1.2	3.2± 1.6	2.4± 2.4

The data are presented as the means ± SD with significance at *p < 0.05, **p < 0.01 and ***p < 0.001 level compared to IBD control.

Supplementary table 2. Dose-dependent effect of ASC-EXO on mouse acute model DAI scoring. DAI score of mice were recorded daily from day -5 to day 10.

Days	DAI Scoring (Average data)				
	Normal	IBD control	ASC-EXO (0.25 x 10^{10} particles/head, 10 inj.)	ASC-EXO (0.5 x 10^{10} particles/head, 10 inj.)	ASC-EXO (1 x 10^{10} particles/head, 10 inj.)
Day-5	0.0 ± 0.0	0.0 ± 0.0	0.0 ± 0.0	0.0 ± 0.0	0.0 ± 0.0
Day-4	0.0 ± 0.0	0.0 ± 0.0	0.0 ± 0.0	0.0 ± 0.0	0.0 ± 0.0
Day-3	0.0 ± 0.0	0.0 ± 0.0	0.0 ± 0.0	0.0 ± 0.0	0.0 ± 0.0
Day-2	0.0 ± 0.0	0.5 ± 0.7	0.1 ± 0.3	0.8 ± 0.4	0.1 ± 0.3
Day-1	0.0 ± 0.0	1.1 ± 1.0	1.1 ± 0.3	1.2 ± 0.4	1.1 ± 0.9
Day0	0.0 ± 0.0	2.5 ± 1.8	2.0 ± 0.9	1.9 ± 0.7	1.8 ± 0.4
Day1	0.0 ± 0.0	5.0 ± 1.8	4.4 ± 0.7	4.5 ± 1.7	4.4 ± 1.4
Day2	0.0 ± 0.0	6.0 ± 1.2	5.4 ± 1.0	5.1 ± 1.4	5.2 ± 1.2
Day3	0.0 ± 0.0	6.1 ± 0.8	4.8 ± 1.1**	4.6 ± 1.3**	4.5 ± 1.8*
Day4	0.0 ± 0.0	5.8 ± 0.8	4.3 ± 1.3**	3.9 ± 2.2*	4.0 ± 1.6**
Day5	0.0 ± 0.0	5.4 ± 1.3	3.9 ± 1.4*	3.4 ± 2.6*	3.3 ± 1.3**
Day6	0.0 ± 0.0	5.0 ± 1.4	3.3 ± 1.6*	2.9 ± 2.3*	2.6 ± 1.9**
Day7	0.0 ± 0.0	4.3 ± 1.5	2.7 ± 1.8*	2.5 ± 2.2*	2.3 ± 1.9*
Day8	0.0 ± 0.0	3.6 ± 1.7	2.2 ± 1.9	2.1 ± 2.2	1.8 ± 1.7*
Day9	0.0 ± 0.0	2.6 ± 1.8	1.7 ± 1.1	1.6 ± 1.8	1.4 ± 1.4
Day10	0.0 ± 0.0	2.0 ± 1.8	1.5 ± 0.8	1.2 ± 1.7	1.2 ± 1.1

The data are presented as the means ± SD with significance at *p < 0.05, **p < 0.01 and ***p < 0.001 level compared to IBD control.

Supplementary table 3. Co-administration effect of ASC-EXO with anti-IL-12 drug on mouse acute model DAI scoring. DAI score of mice were recorded daily from day 0 to day 10.

Days	DAI Scoring (Average data)					
	Normal	IBD control	Ab (Anti-IL-12 p40)	ASC-EXO (1 x 10^{10} particles/head, 10 inj.)	ASC-EXO (1 x 10^{10} particles/head, 10 inj.) + Ab (Anti-IL-12 p40)	ASC-EXO (1 x 10^{10} particles/head, 5 inj.) + Ab (Anti-IL-12 p40)
Day0	0.0 ± 0.0	0.0 ± 0.0	0.0 ± 0.0	0.0 ± 0.0	0.0 ± 0.0	0.0 ± 0.0
Day1	0.0 ± 0.0	0.5 ± 0.5	0.3 ± 0.5	0.2 ± 0.4	0.4 ± 0.7	0.4 ± 0.7
Day2	0.0 ± 0.0	0.3 ± 0.5	0.5 ± 0.8	0.3 ± 0.5	0.9 ± 1.0	0.6 ± 0.7
Day3	0.0 ± 0.0	1.5 ± 1.2	1.0 ± 1.5	0.4 ± 0.5	0.9 ± 0.7	1.3 ± 0.9
Day4	0.0 ± 0.0	1.8 ± 1.0	1.7 ± 1.7	0.9 ± 0.9	1.3 ± 1.1	1.8 ± 0.9
Day5	0.0 ± 0.0	3.3 ± 1.4	3.3 ± 1.6	1.8 ±1.1*	2.5 ± 1.0	3.2 ± 1.0
Day6	0.0 ± 0.0	5.0 ± 1.7	4.7 ± 1.1	3.3 ± 1.5	4.3 ± 1.1	4.8 ± 0.8
Day7	0.0 ± 0.0	6.0 ± 1.1	5.0 ± 0.8	4.3 ± 1.3*	4.4 ± 1.3*	5.1 ± 1.0
Day8	0.0 ± 0.0	5.8 ± 1.6	4.9 ± 1.2	5.0 ± 1.9	3.9 ± 1.2*	4.7 ± 1.4
Day9	0.0 ± 0.0	5.5 ± 1.8	4.6 ± 1.7	4.3 ± 1.9	3.2 ± 1.1*	4.0 ± 1.7
Day10	0.0 ± 0.0	5.3 ± 2.3	3.6 ± 1.8	3.3 ± 2.0	2.6 ± 1.3*	3.4 ± 1.5

The data are presented as the means ± SD with significance at *p < 0.05, **p < 0.01 and ***p < 0.001 level compared to IBD control.

Supplementary table 4. Effects of ASC-EXO IV/IP injection on mouse acute colitis model DAI scoring. DAI score of mice were recorded daily from day 0 to day 10.

Days	DAI Scoring (Average data)		
	IBD control	ASC-EXO (1 x 10^{10} particles/head, IV inj.)	ASC-EXO (1 x 10^{10} particles/head, IP inj.)
Day0	0.0 ± 0.0	0.0 ± 0.0	0.0 ± 0.0
Day1	0.6 ± 0.5	1.1 ± 0.6	1.3 ± 0.5
Day2	0.1 ± 0.3	0.8 ± 0.9	0.5 ± 0.7
Day3	0.8 ± 0.8	0.9 ± 0.6	0.6 ± 0.8
Day4	1.5 ± 1.3	0.3 ± 0.5	0.5 ± 0.5
Day5	3.2 ± 1.9	1.4 ± 1.3*	1.8 ± 1.5
Day6	5.0 ± 1.9	3.1 ± 1.3*	3.3 ± 2.0
Day7	6.6 ± 1.5	3.5 ± 1.6***	4.2 ± 1.6**
Day8	7.1 ± 1.6	4.5 ± 1.6**	5.8 ± 1.9
Day9	6.8 ± 1.9	4.0 ± 1.6**	5.7 ± 2.2
Day10	6.4 ± 2.3	3.5 ±1.7*	5.6 ± 2.0

The data are presented as the means ± SD with significance at *p < 0.05, **p < 0.01 and ***p < 0.001 level compared to IBD control.
